# Supplementary material for: Effectiveness of various bioreactors for thraustochytrid culture and production (Aurantiochytruim limacinum BUCHAXM 122)
Source: PeerJ. 2021 May 27;9:e11405. doi: 10.7717/peerj.11405 (PMC8164841; doi:10.7717/peerj.11405)
Supplement: Supplemental Information 1 — All sparger types were tested, using aeration rate of 3 vvm in bubble, internal loop airlift and stirred tank bioreactor, with an agitation speed of 450 rpm. [file peerj-09-11405-s001.docx]

| **Bioreactor type** | **k_L_a (h^-1^)** | | | | | | |
| --- | --- | --- | --- | --- | --- | --- | --- |
|  |  | **Silicone tube** | **Single coarse-bubble air stone** | **Single fine-bubble air stone** | **Four fine-bubble air stones** | **Four cylindrical super-fine bubble air stones** | **Eight cylindrical super-fine bubble air stones** |
| Shaker 200 rpm | 132.98 ± 18.75 | - | - | - | - | - | - |
| Stirred tank |  | 18.52 ± 0.60 | 52.52 ± 1.35 | 76.34 ± 3.82 | 80.10 ± 4.95 | 99.59 ± 1.65 | 123.66 ± 15.47 |
| Bubble |  | 23.09 ± 0.34 | 50.33 ± 1.31 | 63.13 ± 2.08 | 68.17 ± 3.05 | 106.37 ± 8.92 | 122.99 ± 21.04 |
| Internal loop airlift |  | 31.23 ± 1.39 | 106.63 ± 5.08 | 123.43 ± 4.01 | - | 82.75 ± 1.34 | 123.53 ± 4.57 |
