# Supplementary material for: Effectiveness of various bioreactors for thraustochytrid culture and production (Aurantiochytruim limacinum BUCHAXM 122)
Source: PeerJ. 2021 May 27;9:e11405. doi: 10.7717/peerj.11405 (PMC8164841; doi:10.7717/peerj.11405)
Supplement: Supplemental Information 2 [file peerj-09-11405-s002.docx]

| **Time (h)** | **Shaker**  **200 rpm** | | | **Stirred tank**  **2 vvm** | | | **Bubble**  **2 vvm** | | | **Internal loop airlift**  **1.5 vvm** | | |
| --- | --- | --- | --- | --- | --- | --- | --- | --- | --- | --- | --- | --- |
|  | **Biomass** | **Reducing sugar** | | **Biomass** | **Reducing sugar** | | **Biomass** | **Reducing sugar** | | **Biomass** | **Reducing sugar** | |
| 0 | 3.65 ± 0.35 | | 61.61 ± 1.38 | 3.55 ± 0.49 | | 63.79 ± 2.36 | 3.40 ± 0.28 | | 65.62 ± 0.33 | 3.55 ± 0.07 | | 66.16 ± 0.88 |
| 24 | 17.35 ± 1.48 | | 60.68 ± 1.86 | 33.80 ± 1.70 | | 36.69 ± 1.63 | 3.25 ± 0.07 | | 63.03 ± 2.88 | 18.10 ± 0.14 | | 57.86 ± 5.48 |
| 48 | 29.55 ± 1.77 | | 21.01 ± 1.59 | 43.05 ± 0.35 | | 21.84 ± 2.24 | 27.50 ± 1.56 | | 60.20 ± 4.19 | 35.50 ± 1.84 | | 13.25 ± 3.75 |
| 72 | 31.30 ± 0.28 | | 11.02 ± 1.29 | 33.75 ± 1.63 | | 6.00 ± 0.84 | 29.50 ± 0.57 | | 54.84 ± 3.22 | 36.05 ± 0.92 | | 13.70 ± 3.13 |
| 96 | 31.90 ± 0.71 | | 2.49 ± 1.20 | 30.70 ± 1.84 | | 2.45 ± 0.98 | 34.35 ± 1.48 | | 49.52 ± 3.19 | 36.25 ± 0.78 | | 12.63 ± 5.78 |
| 120 | 32.90 ± 0.28 | | 2.04 ± 0.46 | 30.65 ± 1.20 | | 2.07 ± 0.12 | 38.55 ±4.60 | | 41.87 ± 0.10 | 37.60 ± 3.82 | | 3.61 ± 2.67 |
