# Supplementary material for: Effectiveness of various bioreactors for thraustochytrid culture and production (Aurantiochytruim limacinum BUCHAXM 122)
Source: PeerJ. 2021 May 27;9:e11405. doi: 10.7717/peerj.11405 (PMC8164841; doi:10.7717/peerj.11405)
Supplement: Supplemental Information 3 — Aeration provided at 2 vvm for bubble and stirred tank bioreactors with agitation speed at 600 rpm and 1.5 vvm for internal loop airlift bioreactor. [file peerj-09-11405-s003.docx]

| **Time (h)** | **Fatty acid** | | **Shaker**  **200 rpm** | **Stirred tank** | **Bubble** | **Internal loop airlift** |
| --- | --- | --- | --- | --- | --- | --- |
| 24 | 22:6 (DHA) | mg/g DW | 88.68 ± 0.25^a^ | 130.20 ± 17.22^b^ | - | 158.54 ± 2.49^c^ |
|  |  | % TFA | 28.25 ± 2.53 | 34.71 ± 4.05 | - | 29.10 ± 1.73 |
|  | Others | % TFA | 11.16 ± 3.31 | 3.45 ± 2.16 | - | 6.97 ± 3.08 |
|  | SUM SFA | % TFA | 52.13 ± 3.06 | 54.41 ± 5.03 | - | 57.50 ± 1.84 |
|  | SUM MUFA | % TFA | 1.38 ± 0.15 | 0.58 ± 0.27 | - | 0.75 ± 0.46 |
|  | SUM PUFA | % TFA | 35.33 ± 3.32 | 41.56 ± 5.28 | - | 34.78 ± 1.91 |
| 48 | 22:6 (DHA) | mg/g DW | 113.62 ± 10.10^a^ | 142.00 ± 3.49^b^ | 74.34 ± 5.17^c^ | 175.84 ± 5.01^d^ |
|  |  | % TFA | 29.49 ± 2.29 | 34.47 ± 0.22 | 31.73 ± 1.79 | 30.72 ± 2.34 |
|  | Others | % TFA | 13.47 ± 2.22 | 4.26 ± 0.96 | 3.57 ± 0.77 | 4.59 ± 0.49 |
|  | SUM SFA | % TFA | 50.62 ± 4.06 | 54.50 ± 6.91 | 55.77 ± 5.31 | 58.54 ± 2.27 |
|  | SUM MUFA | % TFA | 0.51 ± 0.28 | 0.55 ± 0.09 | 0.95 ± 0.09 | 0.54 ± 0.06 |
|  | SUM PUFA | % TFA | 35.41 ± 0.54 | 40.70 ± 0.77 | 39.72 ± 2.21 | 36.33 ± 2.84 |
| 72 | 22:6 (DHA) | mg/g DW | 162.65 ± 21.50^a^ | 139.79 ± 16.62^b^ | 129.65 ± 2.29^b^ | 189.95 ± 9.98^c^ |
|  |  | % TFA | 27.73 ± 1.38 | 32.15 ± 4.05 | 37.82 ± 3.38 | 36.93 ± 0.64 |
|  | Others | % TFA | 19.07 ±4.29 | 6.35 ± 1.88 | 5.31 ± 2.93 | 1.02 ± 0.71 |
|  | SUM SFA | % TFA | 47.88 ± 1.54 | 54.18 ± 7.24 | 47.86 ± 7.71 | 55.25 ±2.52 |
|  | SUM MUFA | % TFA | 1.10 ± 0.70 | 0.62 ± 0.07 | 1.27 ± 0.36 | 0.53 ± 0.37 |
|  | SUM PUFA | % TFA | 31.94 ± 2.18 | 38.84 ± 4.22 | 45.56 ± 4.66 | 43.19 ± 1.40 |
| 96 | 22:6 (DHA) | mg/g DW | 190.50 ± 10.64^a^ | 121.50 ± 5.49^b^ | 120.37 ± 1.18^b^ | 189.77 ± 7.65^a^ |
|  |  | % TFA | 35.15 ± 2.31 | 25.68 ± 0.65 | 27.52 ± 0.17 | 38.15 ± 0.70 |
|  | Others | % TFA | 12.19 ± 0.25 | 7.84 ± 1.68 | 6.96 ± 5.78 | 5.63 ± 2.72 |
|  | SUM SFA | % TFA | 45.81 ± 8.21 | 58.38 ± 3.05 | 58.69 ± 4.33 | 48.93 ± 78.99 |
|  | SUM MUFA | % TFA | 0.93 ± 0.51 | 0.59 ± 0.30 | 0.84 ± 0.13 | 0.71 ± 0.27 |
|  | SUM PUFA | % TFA | 41.06 ± 3.20 | 33.19 ± 1.31 | 33.52 ± 1.48 | 44.73 ± 1.82 |
| 120 | 22:6 (DHA) | mg/g DW | 144.16 ± 4.63^a^ | 113.45 ± 1.95^b^ | 119.66 ± 17.45^b^ | 199.02 ± 0.41^c^ |
|  |  | % TFA | 28.60 ± 0.98 | 24.58 ± 0.37 | 33.47 ± 5.20 | 35.36 ± 2.51 |
|  | Others | % TFA | 18.54 ± 4.94 | 11.34 ± 1.55 | 9.75 ± 10.73 | 9.49 ± 7.27 |
|  | SUM SFA | % TFA | 47.08 ± 2.82 | 55.58 ± 2.04 | 48.89 ± 6.08 | 49.11 ± 5.24 |
|  | SUM MUFA | % TFA | 0.86 ± 0.08 | 0.55 ± 0.03 | 0.96 ± 0.11 | 1.07 ± 0.21 |
|  | SUM PUFA | % TFA | 33.52 ± 2.31 | 32.53 ± 0.72 | 40.40 ± 5.68 | 40.32 ± 2.98 |

Means in the same row with different superscript letters are significantly different at p < 0.05; SFA= saturated fatty acids, MUFA = monounsaturated fatty acids, PUFA = polyunsaturated fatty acids.
