# Supplementary material for: Effectiveness of various bioreactors for thraustochytrid culture and production (Aurantiochytruim limacinum BUCHAXM 122)
Source: PeerJ. 2021 May 27;9:e11405. doi: 10.7717/peerj.11405 (PMC8164841; doi:10.7717/peerj.11405)
Supplement: Supplemental Information 4 [file peerj-09-11405-s004.docx]

| **Time (h)** | **Shaker**  **200 rpm** | | | **Stirred tank**  **0.1 vvm** | | | **Bubble**  **0.1 vvm** | | | **Internal loop airlift**  **0.1 vvm** | | |
| --- | --- | --- | --- | --- | --- | --- | --- | --- | --- | --- | --- | --- |
|  | **Biomass** | **Reducing sugar** | | **Biomass** | **Reducing sugar** | | **Biomass** | **Reducing sugar** | | **Biomass** | **Reducing sugar** | |
| 0 | 1.38 ± 0.04 | | 62.28 ± 0.42 | 1.33 ± 0.04 | | 62.74 ± 1.52 | 1.38 ± 0.11 | | 61.24 ± 1.03 | 1.33 ± 0.53 | | 61.16 ± 0.56 |
| 24 | 7.78 ± 0.32 | | 51.79 ± 0.18 | 2.40 ± 0.17 | | 52.04 ± 0.04 | 1.53 ± 0.18 | | 51.19 ± 0.04 | 1.63 ± 0.11 | | 51.74 ± 0.04 |
| 48 | 19.65 ± 2.90 | | 49.65 ± 0.02 | 12.50 ± 0.85 | | 50.56 ± 0.36 | 4.35 ± 0.78 | | 51.37 ±0.07 | 6.86 ± 0.20 | | 51.52 ± 0.13 |
| 72 | 22.35 ± 3.89 | | 47.51 ± 1.68 | 25.00 ± 0.99 | | 49.57 ± 0.46 | 4.77 ± 0.02 | | 51.42 ± 0.14 | 16.20 ± 0.42 | | 50.33 ± 0.78 |
| 96 | 24.40 ± 0.85 | | 25.51 ± 0.07 | 23.91 ± 1.43 | | 25.78 ± 0.43 | 7.70 ± 0.71 | | 50.99 ± 0.25 | 21.40 ± 0.84 | | 35.17 ± 0.40 |
| 120 | 24.08 ± 4.84 | | 13.51 ± 0.21 | 23.30 ± 1.27 | | 13.76 ± 0.79 | 21.15 ± 1.48 | | 53.53 ± 0.12 | 23.41 ± 0.29 | | 25.63 ± 0.28 |
| 144 | 22.35 ± 1.06 | | 13.28 ± 0.22 | 21.80 ± 0.28 | | 13.83 ± 0.71 | 19.90 ± 0.99 | | 45.78 ± 0.08 | 21.75 ± 0.07 | | 13.72 ± 0.84 |
| 168 | 22.00 ± 0.85 | | 13.19 ± 0.05 | 18.50 ± 0.99 | | 13.51 ± 0.18 | 16.55 ± 1.48 | | 38.42 ± 0.06 | 16.50 ± 0.42 | | 13.88 ± 0.79 |
